# Transcriptome analysis of adipose tissues from two fat-tailed sheep breeds reveals key genes involved in fat deposition

**DOI:** 10.1186/s12864-018-4747-1

**Published:** 2018-05-08

**Authors:** Baojun Li, Liying Qiao, Lixia An, Weiwei Wang, Jianhua Liu, Youshe Ren, Yangyang Pan, Jiongjie Jing, Wenzhong Liu

**Affiliations:** 1 Shanxi Agricultural University, College of Veterinary and Animal Science, Taigu, 030801 China; 2 Shanxi Agricultural University, College of Information, Taigu, 030800 China

**Keywords:** Adipose tissue, Fat deposition, Gene expression, RNA sequencing, Sheep

## Abstract

**Background:**

The level of fat deposition in carcass is a crucial factor influencing meat quality. Guangling Large-Tailed (GLT) and Small-Tailed Han (STH) sheep are important local Chinese fat-tailed breeds that show distinct patterns of fat depots. To gain a better understanding of fat deposition, transcriptome profiles were determined by RNA-sequencing of perirenal, subcutaneous, and tail fat tissues from both the sheep breeds. The common highly expressed genes (co-genes) in all the six tissues, and the genes that were differentially expressed (DE genes) between these two breeds in the corresponding tissues were analyzed.

**Results:**

Approximately 47 million clean reads were obtained for each sample, and a total of 17,267 genes were annotated. Of the 47 highly expressed co-genes, *FABP4*, *ADIPOQ*, *FABP5*, and *CD36* were the four most highly transcribed genes among all the known genes related to adipose deposition. *FHC*, *FHC-pseudogene*, and *ZC3H10* were also highly expressed genes and could, thus, have roles in fat deposition*.* A total of 2091, 4233, and 4131 DE genes were identified in the perirenal, subcutaneous, and tail fat tissues between the GLT and STH breeds, respectively. Gene Ontology (GO) analysis showed that some DE genes were associated with adipose metabolism. Kyoto Encyclopedia of Genes and Genomes (KEGG) pathway analysis revealed that PPAR signaling pathway and ECM-receptor interaction were specifically enriched. Four genes, namely *LOC101102230*, *PLTP*, *C1QTNF7*, and *OLR1* were up-regulated and two genes, *SCD* and *UCP-1*, were down-regulated in all the tested tissues of STH. Among the genes involved in ECM–receptor interaction, the genes encoding collagens, laminins, and integrins were quite different depending on the depots or the breeds. In STH, genes such as *LAMB3*, *RELN*, *TNXB*, and *ITGA8,* were identified to be up regulated and *LAMB4* was observed to be down regulated.

**Conclusions:**

This study unravels the complex transcriptome profiles in sheep fat tissues, highlighting the candidate genes involved in fat deposition. Further studies are needed to investigate the roles of the candidate genes in fat deposition and in determining the meat quality of sheep.

**Electronic supplementary material:**

The online version of this article (10.1186/s12864-018-4747-1) contains supplementary material, which is available to authorized users.

## Background

Sheep is a major livestock resource of meat, milk, wool, and fur around the world. Fat-tailed sheep account for approximately 25% of the world’s sheep population [1]. The “fat tail” trait of sheep is regarded as an adaptive response to harsh environment and the fat stored in such tail is a valuable reserve for sheep during migration and in winter when food is scarce [2]. The tail fat is also used as food by humans. However, because too much fat in the daily diet is considered harmful to human health, the carcass adiposity, especially the fat tail, is not desirable to customers and reduces the value of meat in sheep. Therefore, reduction of fat deposition in the body and tail for production of lean meat is a major goal of the sheep industry. This has been attained by docking the fat tail, slaughtering at an early age, or by crossing of the fat-tailed breed with the lean-tailed breed. A better understanding of the molecular mechanism underlying fat deposition is very important for controlling the fat mass in carcass through sheep breeding.

The tail types in the two Chinese domestic fat-tailed sheep breeds, Guangling Large Tailed (GLT) and Small Tailed Han (STH), show great divergence. Both these breeds originated from the ancient Mongolian sheep. The GLT breed is characterized by large tails and good meat quality but shows low fecundity whereas the STH breed typically has small tails and higher fecundity [3]. The difference in the content of tail fat between these two breeds has stimulated extensive interest in characterizing the expression of key genes associated with fat deposition, namely *UCP-1*, *ANGPTL4*, *Lpin2*, and *Lpin3* [3–5]. However, the fat deposition trait is a complex quantitative trait that is controlled by multiple genes. The mechanism responsible for the observed differences in fat deposition between the GLT and STH breed is not yet clear.

The burgeoning developments in the “omics” technologies, such as in trascriptomics, have dramatically changed the approach for investigating the mechanisms underlying metabolism, development, growth, disease resistance in various organisms. In sheep, the transcriptome profiles of tissues including heart, skin, muscle, mammary gland, and adipose tissue, have been determined by deep sequencing [6–11]. Miao et al. [6] analyzed the transcriptome information of subcutaneous adipose tissue between STH and Dorset sheep. Wang et al. [7] investigated the differences in the transcriptome profiles of tail fat tissue between Kazak and Tibetan sheep. It is known that adipose tissues from different anatomical locations are heterogeneous with respect to metabolic activities, functions, and genetic regulation [12–16]. In addition, animal species and breeds can also dictate the characteristics of a given adipose tissue depot [17]. Thus, to better understand the genetic regulation of fat deposition in sheep and to elucidate the differences in fat metabolism between the GLT and STH breed, we used perirenal, subcutaneous, and tail fat tissues from both the breeds to obtain a more comprehensive gene expression profile using RNA sequencing (RNA-seq) technology. We identified some genes that play important roles in fat metabolism. These data provide a valuable theoretical basis for selection of fat deposition trait in sheep breeding.

## Methods

### Adipose tissue collection

The indigenous Chinese sheep breeds, GLT and STH, were raised as described previously [3]. Four healthy 10-month-old male sheep from each breed were slaughtered. The adipose tissues in the perirenal (PEF), subcutaneous (SUF), and tail fat (TAF) were rapidly sampled after death. All the samples were immediately frozen in liquid nitrogen and stored at − 80 °C until subsequent use.

### RNA preparation, cDNA library generation, and sequencing

Total RNA was extracted from the different adipose tissues using Trizol reagent (TaKaRa, USA) according to the manufacturer’s protocol. The concentration and integrity of RNA were evaluated using the 2100 Bioanalyzer (Agilent Technologies, Waldronn, Germany). All the samples had RNA Integrity Number values greater than 7.0 and 28S/18S ratio greater than 1.0. The RNA samples from four independent biological replicates (GLT = 4, STH = 4) for each tissue depot were pooled in equal quantities.

After DNase I treatment, poly (A) mRNA was isolated using oligo (dT) magnetic beads (Invitrogen, Carlsbad, CA, USA) and was fragmented into short fragments in the fragmentation buffer. The mRNA fragments were subsequently used as templates for the synthesis of the first-strand cDNA using random hexamer primers and reverse transcriptase. Following the second-strand cDNA generation, short fragments were added with poly (A) tails and ligated to adaptors. After agarose gel electrophoresis, suitable fragments were selected as templates for PCR amplification. Six paired-end cDNA libraries (three for each breed) were constructed. Finally, sequencing of the libraries was performed using Illumina HiSeq 2000 at the Beijing Genomics Institute (Shenzhen, China).

The raw reads were subjected to quality control through SOAPnuke tool, and low quality reads, including reads with adapters, reads in which unknown bases were more than 10%, and reads in which the percentage of the low quality bases (quality value ≤10) was more than 50%, were filtered. The clean reads, thus obtained, were then aligned against the reference genome and the reference genes of *Ovis aries* (Oar_v3.1, http://www.ncbi.nlm.nih.gov/assembly/GCF_000298735.1) with SOAPaligner/SOAP2 [18] and were annotated.

### Gene expression analysis

The gene coverage and RPKM values were calculated to analyze the gene expression. The gene coverage value is equal to the ratio of the number of bases in a gene covered by the uniquely mapped reads to the total number of bases in that gene. The RPKM means numbers of reads per kilobase of exon model in a gene per million mapped reads [19]. The genes detected in all the six samples were referred to as “co-genes.” A heatmap showing the top recurrently expressed co-genes was generated based on log2 RPKM values using the OmicShare tools, a free online platform for data analysis (www.omicshare.com/tools).

The differentially expressed (DE) genes between the GLT and STH samples in pairwise-comparisons of PEF, SUF, and TAF were determined using the method described by Audic and Claverie [20]. The fold changes (log 2Ratio) were estimated according to the normalized gene expression level in each sample [21, 22]. The false discovery rate (FDR) ≤ 0.001 and the absolute value of log 2Ratio ≥ 1 were used as the threshold to judge the significant differences in gene expression.

### Validation of gene expression using quantitative real-time PCR

The expression levels of eight DE genes related to fat deposition were validated using quantitative real-time PCR (qRT-PCR). These genes included *FABP4* (fatty acid binding protein 4), *SLC27A6* (solute carrier family 27 member 6), *SCD* (stearoyl-CoA desaturase), *THRSP* (thyroid hormone responsive protein), *ALDH1A1* (aldehyde dehydrogenase 1 family member A1), *PPT1* (palmitoyl-protein thioesterase 1), *FABP5* (fatty acid binding protein 5), and *GPI* (glucose-6-phosphate isomerase). The ribosomal protein L 13 gene (*RPL13A*) was used as an internal control [4] because it showed consistent expression. The gene-specific primers were designed by Primer-BLAST available at the National Center for Biotechnology Information (NCBI) website; the sequences of the primers used are listed in Additional file 1. The first-strand cDNA was synthesized using the PrimeScript RT reagent Kit with gDNA Eraser (TaKaRa, Dalian, China).

The qRT-PCR was carried out on a 7500 Fast Real-Time PCR System (Applied Biosystems, Foster, CA, USA) using SYBR Premix Ex Taq II kit (Takara, Dalian, China). The following conditions were used for the amplification: 95 °C for 10 min, followed by 45 cycles of 95 °C for 15 s, 60 °C for 1 min, and a melt curve stage of 95 °C for 45 s, 60 °C for 1 min, and 95 °C for 15 s. The relative expression level of each gene was estimated by the 2^-ΔΔCT^ method [23]. The qRT-PCR analysis was performed in triplicate for each sample.

### Analysis of DE genes

Gene Ontology (GO) analysis, which is used to describe the properties of genes in any organism, was employed to analyze the functions of the DE genes. All the DE genes were mapped to the GO terms in the database (http://www.geneontology.org/). The gene numbers for each term were calculated. Thereafter, a hypergeometric test was employed to find the significantly enriched GO terms for the DE genes. The calculated *p*-value was subjected to Bonferroni Correction [24], taking corrected *P* value ≤0.05 as the threshold for GO terms for all the DE genes.

The pathway-based analysis helps in the understanding of specific functions of the DE genes in certain biological process. The Kyoto Encyclopedia of Genes and Genomes (KEGG) database (http://www.kegg.jp/kegg/pathway.html), which is a major public pathway-related database [25], was used to perform pathway enrichment analysis of the DE genes. The method for calculation was the same as that used in the GO analysis. The P and Q values were returned after the pathway analysis. The pathways with Q value ≤0.05 were considered to be significantly enriched for the DE genes.

## Results

### Sequencing and mapping

We generated six cDNA sequencing libraries using PEF, SUF, and TAF from both the GLT and STH individuals. The libraries were sequenced, and six sets of reads were obtained. All the reads were 90 base pair in length. After filtering the low quality raw reads, a total of 47,445,516, 47,347,456, 47,381,754, 47,344,348, 46,953,510, and 47,047,532 clean reads were obtained from GLTPEF, GLTSUF, GLTTAF, STHPEF, STHSUF, and STHTAF samples, respectively (Table 1). About 76.17–79.43% clean reads were mapped to the sheep genome, and 55.76–63.13% reads were mapped to the known reference genes. Of all the clean reads, 52.46–57.27% reads were mapped perfectly to the sheep reference genome without any mismatch, 64.3–71.43% reads had unique matches, 7–11.86% reads showed multi-position matches, and the total number of unmapped reads was 20.57–23.84%. As for the known sheep reference genes, 42.5–47.45% of the clean reads had 100% match, 51.51–57.22% showed only one match, and the percentage of clean reads with multiple matches was 2.6–5.91%. Unfortunately, 36.87–44.24% reads could not be aligned to any of the reference genes.

**Table 1:** Statistics of total reads and mapped reads

Reads		GLPEF	GLTSUF	GLTTAF	STHPEF	STHSUF	STHTAF
Total	Total reads	47,445,516	47,347,456	47,381,754	47,344,348	46,953,510	47,047,532
	Total base pairs	4,270,096,440	4,261,271,040	4,264,357,860	4,260,991,320	4,225,815,900	4,234,277,880
Matches to genome	Total mapped reads	37,675,194	36,058,604	36,171,524	37,605,664	36,184,083	36,699,831
	Perfect match	26,543,043	24,837,018	25,023,096	27,114,486	25,768,571	25,233,708
	≤ 5 bp mismatch	11,132,151	11,221,586	11,148,428	10,491,178	10,415,512	11,466,123
	Unique match	32,990,142	30,442,820	30,831,328	33,818,446	32,895,221	31,906,341
	Multi-position match	4,685,052	5,615,784	5,340,196	3,787,218	3,288,862	4,793,490
	Total unmapped reads	9,770,322	11,288,852	11,210,230	9,738,684	10,769,427	10,347,701
Matches to genes	Total mapped reads	26,559,610	26,402,159	27,534,197	26,776,917	28,230,599	29,701,546
	Perfect match	20,476,006	20,123,960	20,965,259	20,683,554	21,641,557	22,323,002
	≤ 5 bp mismatch	6,083,604	6,278,199	6,568,938	6,093,363	6,589,042	7,378,544
	Unique match	24,440,852	25,169,009	26,230,487	24,656,176	26,501,874	26,918,689
	Multi-position match	2,118,758	1,233,150	1,303,710	2,120,741	1,728,725	2,782,857
	Total unmapped reads	20,885,906	20,945,297	19,847,557	20,567,431	18,722,911	17,345,986

### General gene expression and annotation

**Fig. 1:**
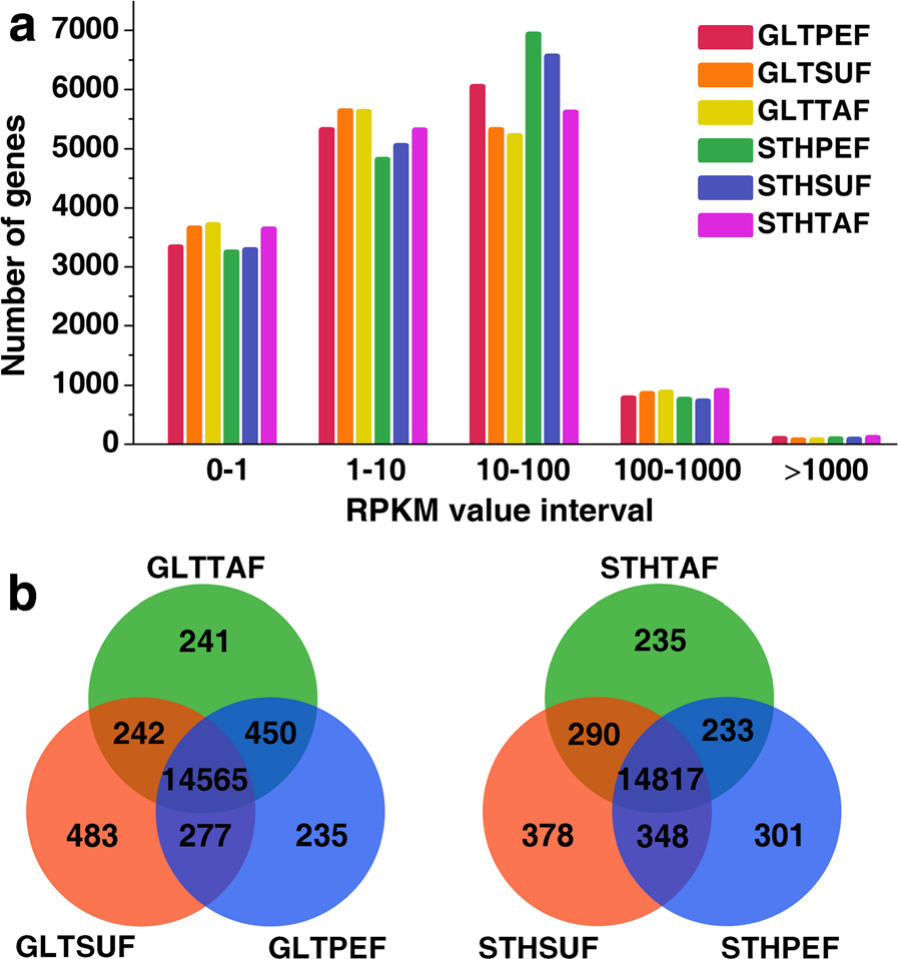
**a** The numbers of annotated genes with different expression levels against the range of RPKM values. **b** Venn diagrams of genes among different fat depots in GLT or STH sheep

The gene coverage was calculated as the percentage of a gene covered by the reads. Among the six libraries, approximately 60% of the reference genes had 90–100% coverage, and 10% or 11% of the annotated genes had 80–90% coverage (Additional file 2). Similar gene coverage was observed in all the RNA-seq libraries. A total of 17,267 annotated genes with RPKM > 0 were detected in all the tested tissues (Additional file 3). Interestingly, the number of genes found among all the samples was very similar with respect to the RPKM value (Fig. 1a). In all the six samples, 82.14% (14,183/17267) of these genes were detected, and 92.78% (16,021/17267) genes were common in both the GLT and STH breeds (Additional file 4). In terms of the breeds, 95.52% (16,493/17267) of the total genes were detected in GLT, and a similar percentage, of 96.15% (16,602/17267) of the total genes, was detected in STH. A total of 472 and 581 genes were exclusively detected in GLT and STH, respectively. In GLT sheep, 15,567, 15,527, and 15,498 genes were detected in SUF, PEF, and TAF, respectively, and 14,565 genes were found to be common in all the three tissues. Similarly, 15,833, 15,699, and 15,575 genes were examined in all the corresponding fat tissues of STH, respectively, and 14,817 genes were detected in all of them. A total of 235, 483, and 241 genes were discovered specifically in PEF, SUF, and TAF of GLT sheep, respectively (Fig. 1b). The number of genes that were uniquely expressed in the corresponding tissues of STH was 301, 378, and 235, respectively.

### Validation of RNA-seq results by qRT-PCR

**Fig. 2:**
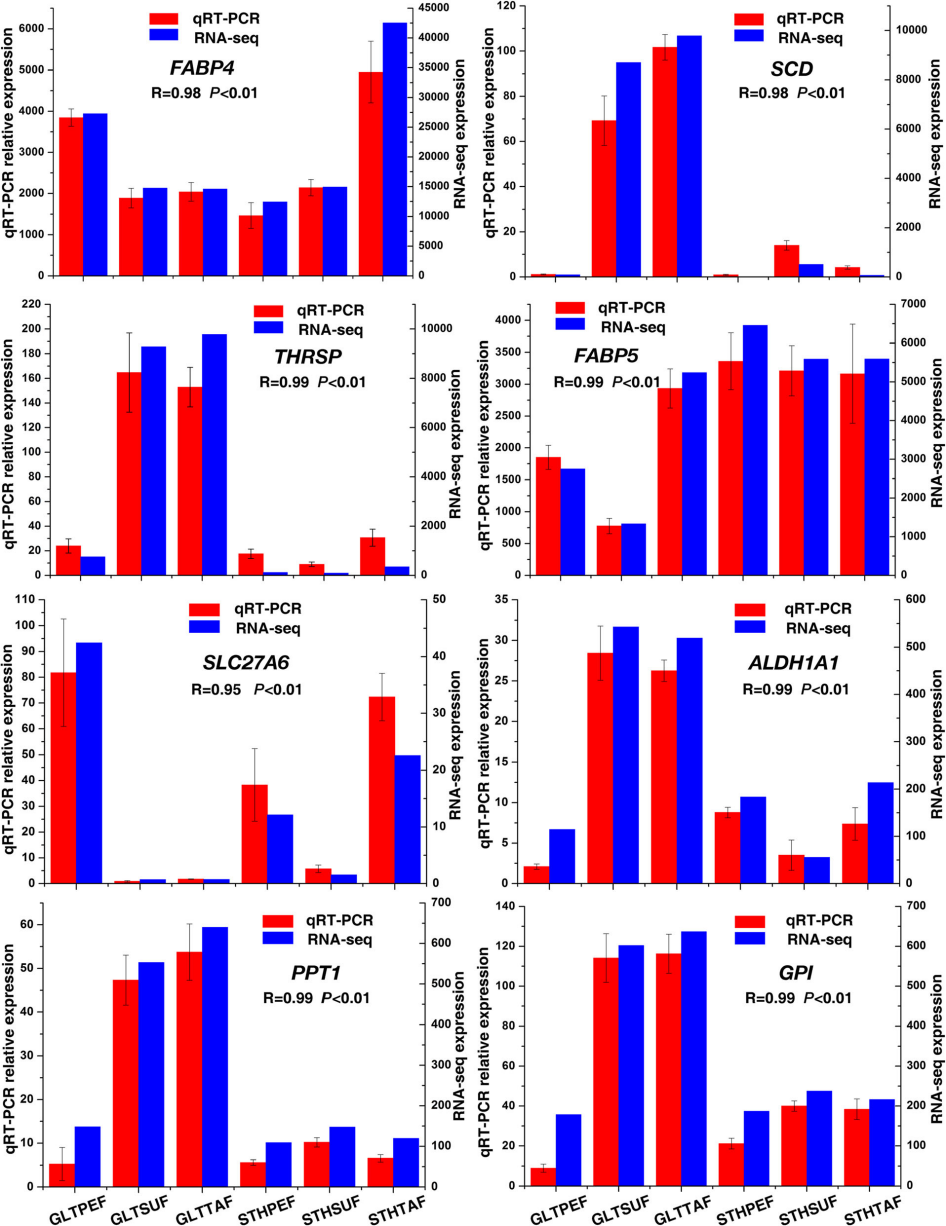
Expression levels of eight genes from qRT-PCR and RNA-seq. The X axis represents the different adipose tissues from both sheep breeds. The Y axis on the left represents the relative gene expression levels of qRT-PCR by columns and bars. The Y axis on the right represents the relative values of RPKM by lines

To validate the RNA-seq results, eight DE genes related to fat deposition were selected for qRT-PCR analysis. We found that the expression patterns of these genes were consistent between the two methods. The Spearman’s correlation coefficient suggested that the data obtained from RNA-seq had a highly significant correlation with that obtained from qRT-PCR (Fig. 2). These results suggested that the expression profile determined by RNA-seq was reliable.

### Analysis of highly expressed co-genes

**Fig. 3:**
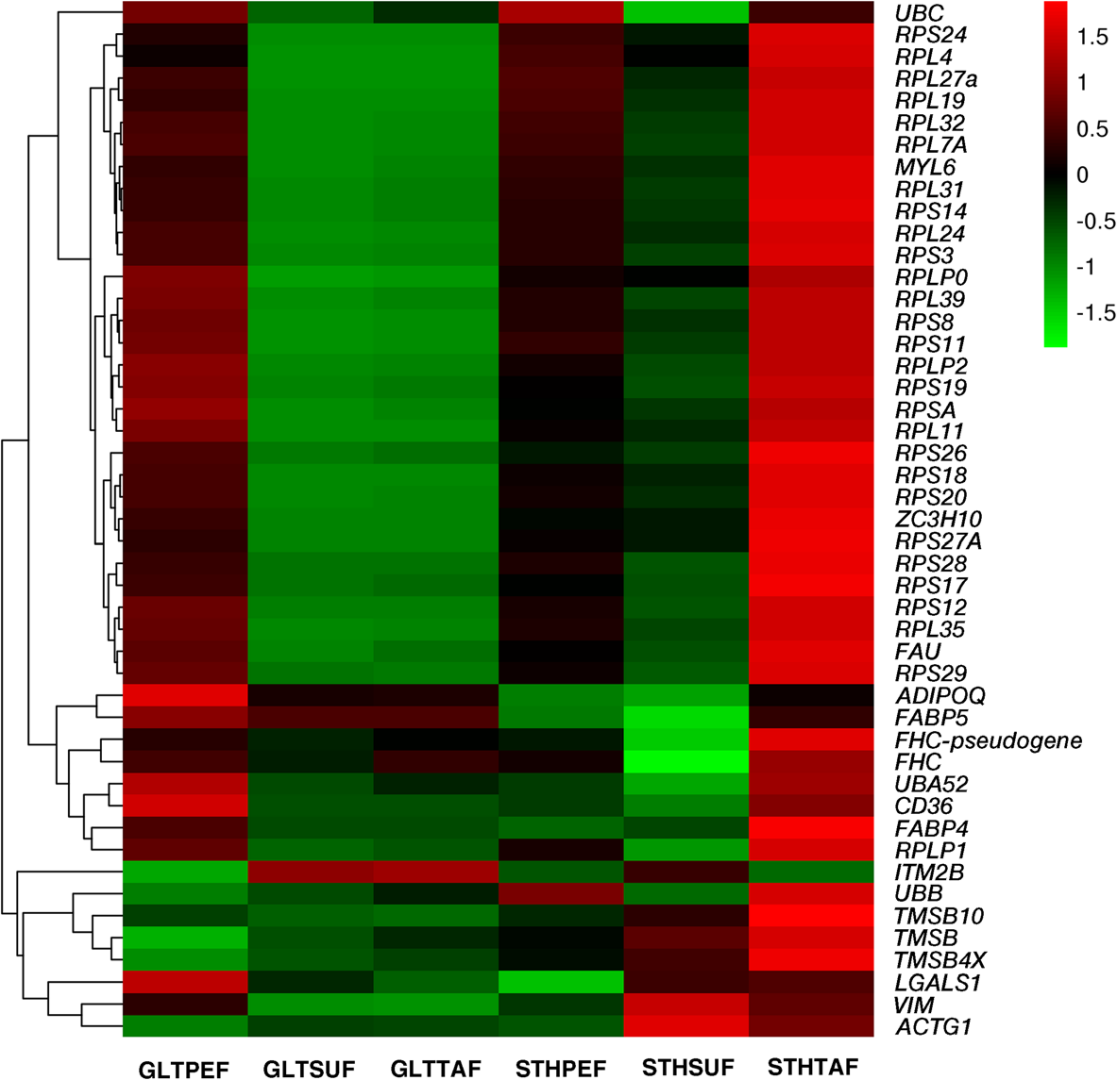
A heat-map showing the top 47 highly expressed co-genes. The cluster analysis of gene expression is based on log2 RPKM data. The red color represents higher expression and the green color represents lower expression

To further illustrate the specific gene expression patterns in the adipose tissues, we selected 47 genes that recurrently appeared in the top 100 most-highly expressed co-genes in each sample, and grouped them into three major clusters based on the RPKM value (Additional file 5 and Fig. 3). The first group consisted of 28 genes, which encoded ribosomal proteins or ribosomal protein homologue (the *FAU* gene), as well as *MYL*, *ZC3H10*, and *UBC*. Several genes related to fat deposition, namely *ADIPOQ*, *FABP5*, *CD36*, and *FABP4*, formed the second group, along with other genes (*FHC*, *FHC-pseudogene*, *UBA52*, and *RPLP1*). Eight genes (*ITM2B*, *UBB*, *TMSB10*, *TMSB*, *TMSB4X*, *LGALS1*, *VIM*, and *ACTG1*) with diverse functions formed another group.

In the heatmap, different colors represent the different expression levels. The red color represents higher expression, and the green color represents lower expression. In the first group, all the genes except *UBC* showed similar differential expression pattern between the two breeds (GLTPEF vs. STHPEF, GLTSUF vs. STHSUF, and GLTTAF vs. STHTAF). Especially in TAF, the ribosomal protein genes had stronger expression in STH than in GLT. Most of the genes from the other two groups exhibited a higher level of expression in STHTAF than in GLTTAF. *FABP4* had the highest mRNA expression level in all the tested fat tissues. There was no obvious difference in the expression in STHTAF vs. GLTTAF for *ADIPOQ* and *FABP5*.

### Expression of DE genes in GLT and STH sheep

**Fig. 4:**
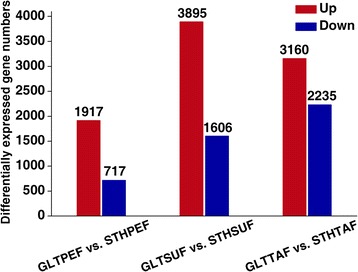
The number of up or down regulated genes in STH as compared to GLT sheep

To gain a global perspective on the differences in gene expression in the GLT and STH breeds, we performed the following pairwise comparisons: GLTPEF vs. STHPEF, GLTSUF vs. STHSUF, and GLTTAF vs. STHTAF. The DE genes were screened using the filtering criteria for the FDR value ≤0.001 and the absolute value of log 2Ratio ≥ 1 (Additional file 6). In the comparisons of PEF, SUF, and TAF for both the breeds, we found 1917 up-regulated and 717 down-regulated genes, 3895 up-regulated and 1606 down-regulated genes, and 3160 up-regulated and 2235 down-regulated genes, respectively (Fig. 4).

**Fig. 5:**
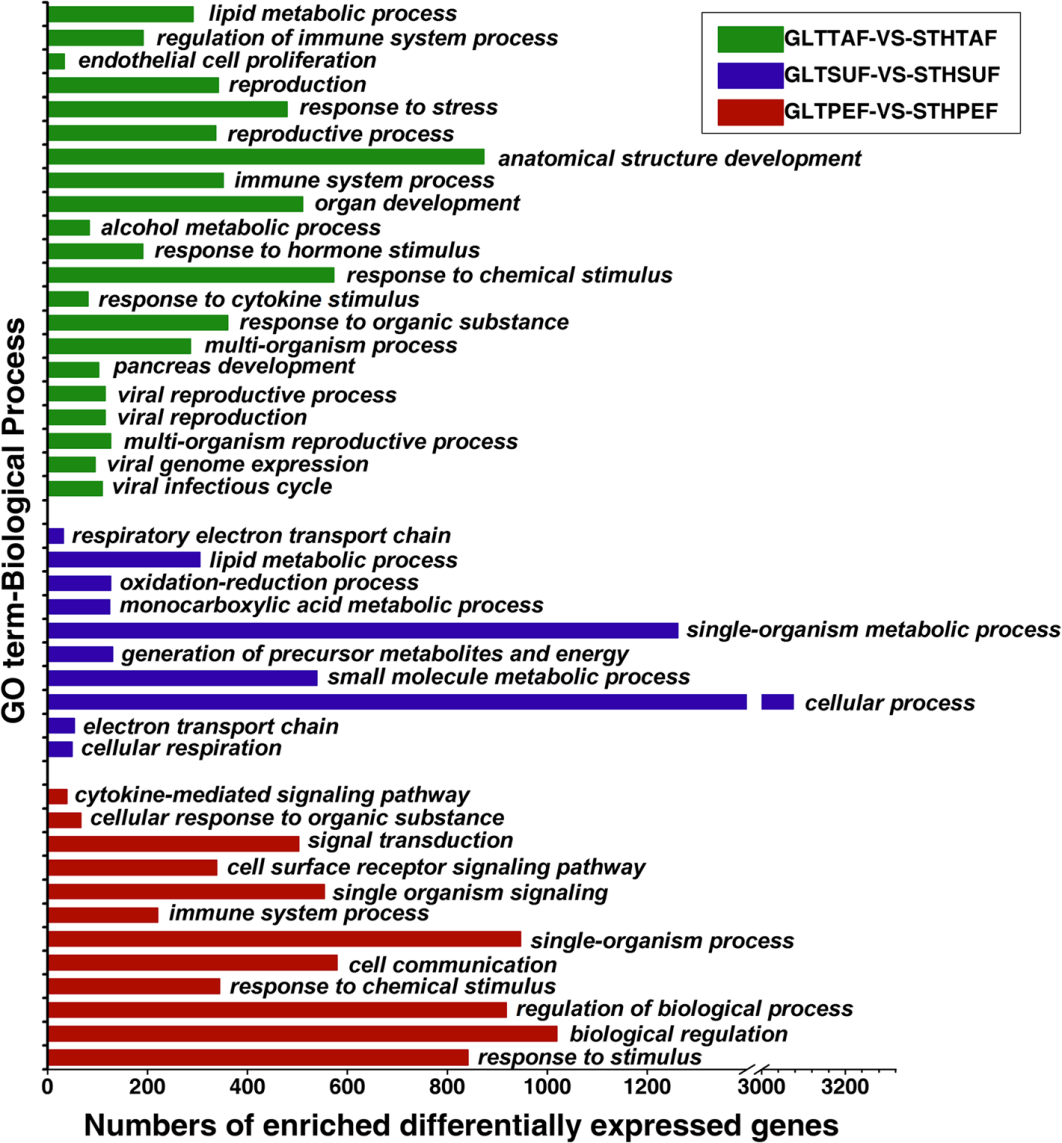
The significantly enriched GO biological process terms of DE genes

The DE genes were classified by GO enrichment based on cellular component, molecular function, and biological process. The results of the selected significant (corrected *p*-value ≤0.05) GO annotation are presented in Additional file 7. Among the variety of cellular component terms, the category of extracellular matrix or extracellular region part was significantly enriched in all the three comparisons. For molecular function, binding related terms were the abundant categories. The term “lipid metabolic process” was also involved in the SUF and TAF comparisons. The top enriched biological process terms are represented in Fig. 5.

To identify the biological pathways that were involved in fat deposition, the DE genes from the three comparisons were mapped to the KEGG pathway database (Additional file 8). The pathways with the Q value ≤0.05 were thought to be significantly enriched. Several pathways related to lipid metabolism were found. In the PEF comparison, the PPAR signaling pathway (33 genes, Q = 0.002916), glycerolipid metabolism (23 genes, Q = 0.006579), Type II diabetes mellitus (20 genes, Q = 0.01202), and adipocytokine signaling pathway (25 genes, Q = 0.03863) were identified. The terms, ECM–receptor interaction (47 genes, Q = 0.00009957274), cell adhesion molecules (59 genes, Q = 0.000004805), and valine, leucine, and isoleucine degradation (18 genes, Q = 0.004779), were also significantly enriched, suggesting that these processes might contribute to fat deposition or fatty acid metabolism.

In GLTSUF vs. STHSUF, the significantly enriched pathways contained the PPAR signaling pathway (55 genes, Q = 0.003290), valine, leucine, and isoleucine degradation (33 genes, Q = 0.009917), Type II diabetes mellitus (33 genes, Q = 0.01313), lipoic acid metabolism (7 genes, Q = 0.04033), and ECM–receptor interaction (68 genes, Q = 0.01587673) pathways. For TAF comparison, the significant pathways did not include the pathways related to fat metabolism; however, Insulin signaling pathway (74 genes, Q = 0.07256), PPAR signaling pathway (49 genes, Q = 0.08449), and ECM–receptor interaction (62 genes, Q = 0.1917799) pathways were among the top enriched pathways.

Together, the PPAR signaling pathway and ECM–receptor interaction were identified as the common pathways with top rank in all the three comparisons. Therefore, we focused on these two pathways for further characterization. The DE genes enriched in the both pathways are listed in Table 2. In the PPAR signaling pathway, a total of nine DE genes, including four up-regulated genes (*LOC101102230*, *PLTP*, *C1QTNF7*, and *OLR1*) and two down-regulated genes (*SCD* and *UCP-1*) were commonly annotated in all the three comparisons (Table 3).

**Table 2:** The annotated differentially expressed genes enriched in the PPAR signaling pathway or ECM–receptor interaction

Pathway	Up-down regulation	GLTPEF vs. STHPEF	GLTSUF vs. STHSUF	GLTTAF vs. STHTAF
PPAR signaling pathway	Up	*LOC101114861*, *LOC101102230*, *CRABP1*, *PLTP*, *SCD5*, *FADS2*, *ACSBG2*, *OLR1*, *CPT1C*, *C1QTNF3*, *COL8A1*, *C1QTNF7*, *LOC101116326*, *CBLN3*, *LOC101114586*, *PDLIM2*	*LOC101102230*, *LOC101113077*, *SLC27A1*, *SLC27A5*, *SLC27A6*, *PLTP*, *OLR1*, *CPT1A*, *CPT1B*, *ANGPTL4*, *COL8A1*, *C1QTNF3*, *COL8A2*, *C1QTNF7*, *CBLN3*, *CAPRIN2*, *C1QTNF5*, *C1QTNF2*, *NEBL*, *PDLIM2*, *SORBS3*	*LOC101102230*, *LOC101113339*, *LOC101113077*, *SLC27A6*, *SLC27A5*, *PLTP*, *SCD5*, *LOC101102230*, *OLR1*, *CPT1B*, *CPT1C*, *CPT1A*, *ANGPTL4*, *PLIN2*, *FABP4*, *C1QTNF7*, *LOC101116326*, *CAPRIN2*, *C1QTNF5*, *LOC101114586*, *NEBL*, *PDLIM2*
	Down	*LOC101113339*, *FABP4*, *FABP5*, *SLC27A6*, *RXRG*, *SCD*, *ANGPTL4*, *PLIN1*, *PLIN5*, *ADIPOQ*, *UCP1*	*FABP5*, *CRABP2*, *CRABP1*, *SLC27A2*, *RXRA*, *SCD*, *FADS2*, *CYB5A*, *ACSL6*, *ACSL5*, *ACSL1*, *ACSBG1*, *PLIN2*, *PLIN5*, *PLIN3*, *ADIPOQ*, *UCP1*	*FABP12*, *CRABP2*, *RXRG*, *RXRA*, *SCD*, *FADS2*, *CYB5A*, *ACSL6*, *ACSL1*, *ACSL5*, *ACSL3*, *LOC101117914*, *SORBS1*, *UCP1*
ECM-receptor interaction	Up	*COL4A6*, *COL4A5*, *COL4A3*, *COL23A1*, *ARID5A*, *COL1A1*, *COL13A1*, *COL1A2*, *LAMB3*, *MEGF9*, *LAMA5*, *RELN*, *THSD1*, *ISM1*, *FN1*, *ITGA5*, *TNC*, *ANGPTL1*, *TNXB*, *TENM3*, *ITGA8*, *ITGA9*, *ITGA11*, *ZAN*, *FCGBP*, *ITGBL1*, *ITGB4*	*COL6A5*, *COL6A6*, *COL6A1*, *COL6A3*, *COL6A2*, *PIMREG*, *KRBA1*, *TNKS1BP1*, *ARID5A*, *EHBP1L1*, *AHNAK*, *LAMB3*, *LAMA2*, *LAMB1*, *RELN*, *THBS1*, *COMP*, *THBS2*, *THBS3*, *THBS4*, *THSD1*, *FN1*, *ITGA5*, *SDK2*, *ANGPTL6*, *ANGPTL1*, *TNXB*, *TNC*, *IGDCC4*, *ITGA8*, *ITGA9*, *ITGA11*, *ITGB3*, *ITGBL1*, *ITGA2*, *ITGA4*, *SPP1*, *PRG4*, *ITGB7*, *ITGB5*	*COL9A3*, *COL2A1*, *COL13A1*, *COL11A2*, *TNKS1BP1*,*COL6A5*, *COL23A1*, *EHBP1L1*, *LAMB3*, *LAMB1*, *LAMA2*, *RELN*, *THBS2*, *THBS1*, *SCARF1*, *TNXB*, *ITGA8*, *VWF*, *ITGA2*, *SPP1*, *VTN*, *TGB7*, *ITGB5*
	Down	*WFIKKN2*, *WAP*, *PIMREG*, *LAMB4*, *COMP*	*COL24A1*, *GRTP1*, *COL11A1*, *LOC101117707*, *LAMB4*, *SSPO*, *C5H19orf70*, *ITGA3*	*COL24A1*, *PIMREG*, *GRTP1*, *COL5A1*, *COL3A1*, *COL5A3*, *COL5A2*, *COL11A1*, *COL1A2*, *COL1A1*, *LAMC2*, *LAMB4*, *LOC101117707*, *MEGF9*, *ATRN*, *SM1*, *LOC101120408*, *TENM4*, *TENM1*, *ITGA1*, *ITGA6*, *TGAV*, *ITGB8*

The differentially expressed genes (DE genes) were generated from comparisons, GLTPEF vs. STHPEF, GLTSUF vs. STHSUF, and GLTTAF vs. STHTAF. The PPAR signaling pathway and ECM–receptor interaction were identified through KEGG pathway analysis. “Up-down regulation” means that the genes were up- or down-regulated in STH as compared to their expression levels in GLT sheep

**Table 3:** The common differentially expressed genes enriched in PPAR signaling pathway or ECM–receptor interaction

Pathway	Gene information from NCBI website			Expression ration		
	Gene ID	Gene name	Gene annotation	STHPEF/GLTPEF	STHSUF/GLTSUF	STHTAF/GLTTAF
PPAR signaling pathway	101,102,230	*LOC101102230*	platelet glycoprotein 4-like, FATCD36	Up	Up	Up
	101,115,982	*PLTP*	phospholipid transfer protein	Up	Up	Up
	101,117,918	*OLR1*	oxidized low density lipoprotein receptor 1	Up	Up	Up
	101,105,159	*C1QTNF7*	C1q and tumor necrosis factor related protein 7	Up	Up	Up
	101,104,743	*SLC27A6*	solute carrier family 27 member 6	Down	Up	Up
	101,104,317	*ANGPTL4*	angiopoietin like 4	Down	Up	Up
	101,108,615	*FADS2*	fatty acid desaturase 2	Up	Down	Down
	443,185	*SCD*	stearoyl-CoA desaturase	Down	Down	Down
	494,434	*UCP-1*	uncoupling protein 1	Down	Down	Down
ECM-receptor interaction	101,105,176	*LAMB3*	laminin subunit beta 3	UP	UP	UP
	101,120,658	*RELN*	reelin	UP	UP	UP
	100,820,740	*TNXB*	tenascin XB	UP	UP	UP
	101,117,689	*ITGA8*	integrin subunit alpha 8	UP	UP	UP
	101,106,520	*LAMB4*	laminin subunit beta 4	Down	Down	Down

The differentially expressed (DE) genes listed in this table were common in the comparisons, GLTPEF vs. STHPEF, GLTSUF vs. STHSUF, and GLTTAF vs. STHTAF. The PPAR signaling pathway and ECM–receptor interaction were enriched through KEGG pathway analysis. The gene expression level was evaluated by RPKM. The ratio was defined as the RPKM value of a gene in one tissue of STH to that in the corresponding tissue of GLT

In the ECM–receptor interaction, the collagen genes (for example, *COL4A6*, *COL6A5*, and *COL9A3*), laminin genes (for example, *LAMB3*, *LAMB4*, and *LAMA2*), and Integrin genes (for example, *ITGA5*, *ITGA9*, and *ITGA1*) were differentially expressed between the two breeds (Table 2). The Collagen IV genes (*COL4A6*, *COL4A5*, and *COL4A3*) were up-regulated in GLTPEF. The Collagen VI genes (*COL6A5*, *COL6A6*, *COL6A1*, *COL6A3*, and *COL6A2*) were up-regulated in GLTSUF. However, Collagen V genes (*COL5A1*, *COL5A3*, and *COL5A2*) were up-regulated in STHTAF. Among the common genes involved in the ECM–receptor interaction, five genes (*LAMB3*, *RELN*, *TNXB*, and *ITGA8*) were differentially up-regulated in STH, and *LAMB4* was down-regulated (Table 3).

## Discussion

The mechanisms of fat metabolism are complex, and manipulation of fat deposition for meat production is very important in sheep breeding. Crossing of sheep breeds with different patterns of fat deposition is one of the agronomical strategies for improving the meat quality. Previous studies have investigated the differences in gene expression patterns or molecular genetic mechanisms between characteristically different sheep breeds [3, 5, 6, 26–28]. To investigate the genetic profiles of fat tissues and to understand the differences in the genetic mechanisms determining fat deposition between distinct breeds, we characterized the transcriptome of PEF, SUF, and TAF from GLT and STH sheep breeds.

The qRT-PCR analysis showed that the transcriptome profiles determined by RNA-seq were reliable. The percentage of reads that could be mapped to the reference genome of sheep was comparable to that reported for pigs [29, 30] and cattle [31, 32], where 60.2–78.3% of the reads could be mapped to the respective reference genomes. The percentage of reads mapped to the reference genes were lower than that mapped to the sheep genome, owing to the GC content, type of cells, and for other reasons, as described earlier [33, 34]. It should be noted that the percentage of total reads not mapped to the reference genome (20.57–23.84%) or to the reference genes (36.87–44.24%) was not low. This could be caused by imperfections in the reference genome, reference errors, sequencing errors, and the defined mapping criteria [35].

The identification of co-genes transcribed in all the tested fat tissues and the genes that were differentially expressed between the two breeds not only reveals the possible mechanism controlling fat deposition in sheep and the possible novel functions of known genes, but also provides valuable information for understanding the phenotypic and functional differences in the deposition of fat in livestock.

### Top highly expressed candidate genes for fat deposition in sheep

We found four candidate genes for fat deposition, namely *FABP4*, *CD36*, *FABP5*, and *ADIPOQ*, which were abundantly expressed in the fat tissues of sheep. *FABP4* is thought to play roles in fatty acid transport and fat deposition in animals as well as in human metabolic syndrome [36]. Previous studies have shown that *FABP4* is involved in fat accumulation in cattle [37] and in determining the tenderness of meat in sheep [38]. *CD36* can bind long chain fatty acids and plays an important role in the absorption and storage of dietary lipids [39, 40]. *FABP5* has similar roles as *FABP4* and could compensate for the loss of *FABP4* in adipocytes [41]. *ADIPOQ* (Adiponectin), an important adipocytokine that is secreted by adipocytes, modulates the regulation of glucose and fatty acid oxidation [42–44]. Polymorphisms in *ADIPOQ* have been suggested to be associated with fat deposition and carcass traits in pigs [45], and with meat marbling in cattle [46, 47]. The analysis of the Sheep Quantitative Trait Locus (QTL) Database (Sheep QTLdb: https://www.animalgenome.org/cgi-bin/QTLdb/OA/index) showed that the chromosomal location of *ADIPOQ* is within the QTL region for “carcass fat percentage.” This suggested that *ADIPOQ* also might be associated with the sheep fatness trait. Strong transcription of these four genes observed in the present study confirms their extreme importance in adipose deposition in sheep.

FHC (Ferritin heavy chain) is one of the subunits of Ferritin, a ubiquitous intracellular protein that stores iron [48]. Previous studies have reported the up-regulation of *FHC* by adiponectin in skeletal muscle cells [49] and a consistent increase in *FHC* expression during the differentiation of 3 T3-L1 preadipocytes [50]. Our results suggest that *FHC* and *FHC pseudogene* express abundantly in sheep fat tissues, and the two genes were found to cluster together with other genes related to fat deposition. Genes with similar expression pattern could be grouped together through clustering analysis, suggesting that such genes might have similar functions. Therefore, we infer that the two FHC related genes are closely associated with fat cell activity. Another candidate gene for fat deposition, *ZC3H10* (Zinc Finger CCCH-Type Containing 10), was reported to be down-regulated in breast cancer cells [51] and is expected to have a tumor suppressor function [52]. The finding on the chromosomal location of *ZC3H10* within the QTL region for “internal fat amount” obtained from the Sheep QTLdb indicated its association with fat deposition in sheep. However, its role in adipocytes has not been studied. Further investigation is required to determine whether or not these genes are involved in adipocyte homeostasis.

### Several DE genes are responsible for fat deposition in sheep

The PPAR signaling pathway regulates cellular differentiation, energy balance, and lipid metabolism [53]. In the present study, multiple genes enriched in PPAR signaling pathway were found to be differentially regulated in the tissues of the two sheep breeds (Table 3). *PLTP*, *OLR1*, *C1QTNF7*, and *LOC101102230* were up-regulated in the STH breed. It is known that PLTP (phospholipid transfer protein) is involved in transferring surface lipids from triglyceride-rich lipoproteins to HDL during lipolysis [54]. OLR1 (Oxidized low-density lipoprotein receptor 1) is expressed in multiple cell types, including adipocytes [55]. It can bind to and degrade oxidized low-density lipoproteins. Recent studies have demonstrated that *OLR1* is closely linked to obesity [56], and it was found to be highly expressed in fat pigs compared to lean animals [57]. There are evidences that show that SNPs in *PLTP* [58] or *OLR1* [59, 60] are significantly associated with economic traits, such as marbling score, in beef. C1QTNF7 (C1q and tumor necrosis factor related protein 7), also called CTRP7, shares a high structural similarity with adiponectin. It is assumed that C1QTNF7 mimics the effects of adiponectin and is activated by PPAR. The *C1QTNF7* transcripts were found to be expressed predominantly in the adipose tissue [61], and deletion of *C1QTNF7* attenuated obesity-linked glucose intolerance, adipose tissue inflammation, and hepatic stress [62]. The mRNA sequence of *LOC101102230* (Accession No.: XM_012176565) showed 91% identity with the sequence of sheep *CD36* (Accession: XM_012176587), suggesting that *LOC101102230* might exert roles similar to those of *CD36* in lipid absorption. At present, little is known about *LOC101102230*, and further investigation should be conducted to explore its possible role in fat tissues. Both *SCD* and *UCP-1* were up-regulated in GLT sheep. SCD (Stearoyl-CoA desaturase) is a key enzyme that catalyzes a rate-limiting step in the synthesis of unsaturated fatty acids [63]. The elevated *SCD* activity is positively correlated with increased fat accumulation and monounsaturation of saturated fatty acids [64]. Thus, SCD is considered to be a principle gene in animal breeding that can improve the meat quality by modulating the fat deposition and saturated fatty acid content. The association of the SNPs in *SCD* and meat quality traits in beef [64], pig [65], and sheep [26] has been reported extensively. In this study, much higher mRNA concentrations of *SCD* were detected in SUF than in TAF for both the sheep breeds. The expression pattern of *SCD* observed in this study suggests lower content of saturated fatty acids in GLT compared to that in STH. *UCP1* (the uncoupling protein 1) is found in brown adipose tissues and plays a pivotal role in thermogenesis and in the regulation of lipolysis [66]. The polymorphisms in *UCP1* were found to be associated with milk quality in dairy cows [67] or with carcass traits in sheep [68]. Large tail shape and high mount fat mass present in GLT sheep could partially contribute to the higher expression levels of *SCD* and *UCP1*.

The other genes, such as *ANGPTL4*, *FASD2*, and *SLC27A6*, also behave differently between GLT and STH. The ANGPTL4 (angiopoietin like 4) gene showed a dramatic increase in transcription during and after adipocyte differentiation [69]. Recent research suggests that ANGPTL4 reduces the LPL protein in adipocytes by promoting its intracellular degradation [70]. The delta-6 desaturase encoded by *FASD2* (fatty acid desaturase 2) is one of the important enzymes in the endogenous formation of long-chain polyunsaturated fatty acids. Genetic variation in human *FADS2* was associated with the activity of the desaturation–elongation pathway, whole-body fat oxidation [71], and inflammation of adipose tissue [72]. The SNPs of *ANGPTL4* [73] or *FADS2* [74] showed significant effects on the quality of pig meat. *SLC27A6* (the solute carrier family 27A) is involved in the translocation of long-chain fatty acids across the plasma membrane [75]. A recent study showed that there was significant association of *SLC27A6* polymorphisms with milk quality [76]. *ANGPTL4*, *FASD2*, and *SLC27A6* might play roles in the differences observed in fat metabolism between the two sheep breeds.

### Roles of extracellular matrix in adipose tissues

In adipose tissue, adipocytes are embedded in the extracellular matrix (ECM) network, which predominantly consists of collagen [77]. The ECM provides structural support and anchorage for adipocytes and regulates adipogenesis. Some of the genes that were differentially expressed between GLT and STH sheep were enriched in the ECM–receptor interaction pathway. Different collagens make up the different ECM components; for example, collagen 1, 3, and 5 form the fibrils, collagen 6 forms the microfibril, and collagen 4 forms the basal membrane. Collagen 1 and 3 are secreted by preadipocytes during the early stage of adipocyte differentiation. The levels of collagen 4, 5, and 6 peak at the middle stage of adipocyte differentiation [78]. In the present research, the differential expression of *COL4* in PEF, *COL6* in SUF, and *COL5* in TAF suggests structural distinction of ECM or adipogenetic difference based on the position of the fat depots. Some minor collagen genes, including *collagen 2, 11, 13, 23,* and *24*, were also expressed differentially, which also indicates the complicated histological differences in the adipose tissues. The different expression of other ECM-related genes, like *LAMB3*, *LAMB4*, *ITGA8*, *RELN*, and *TNXB*, also shows the difference in the ECM structure or adipogenic signaling between GLT and STH breeds.

## Conclusions

This study provides a global view of the transcriptome based on three different fat tissue depots from two fat-tailed sheep breeds. The highly transcribed co-genes were identified and the DE genes between these two breeds were analyzed through GO and KEGG database. Our data showed that *FABP4*, *ADIPOQ*, *FABP5,* and *CD36* were related to fat deposition, and were transcribed at very high levels. Nine fat deposition related genes (*LOC101102230*, *PLTP*, *C1QTNF7*, *OLR1*, *SCD*, *UCP-1*, *ANGPTL4*, *FASD2*, and *SLC27A6*) and five ECM related genes (*LAMB3*, *RELN*, *TNXB*, *ITGA8*, and *LAMB4*) might be responsible for the difference in fat deposition between the GLT and STH sheep breeds. Although the RNA samples used for sequencing were obtained by pooling equal amounts of RNA from four independent biological replicates, the qRT-PCR validation confirmed the reliability of our results. The deep sequencing results might need to be validated using more biological replicates. Further studies are required to investigate the roles of the candidate genes in fat deposition for improvement of sheep breeding programs.

## Additional files

Additional file 1: **Table S1.** Primers used in qRT-PCR. (XLS 19 kb)

Additional file 2: **Figure S1.** Gene coverage distribution of the six adipose tissue transcriptomes. Gene coverage is calculated as the percentage of a gene covered by reads. This value is equal to the ratio of the base number in a gene covered by unique mapping reads to the total base number of coding region in that gene. Pies with different colors represent proportions of genes with certain coverage. For example, green pie is indicating proportion of genes with coverage between 90 and 100%. (TIF 7235 kb)

Additional file 3: **Table S2.** All the annotated genes in the six libraries. Unique reads are reads aligned to only one position in the reference sequence. Gene coverage and RPKM values were calculated to analyze gene expression. (XLSX 3230 kb)

Additional file 4: **Table S3.** Summary of the number of genes (RPKM> 0) detected in the six adipose tissues of two breeds. (XLS 20 kb)

Additional file 5: **Table S4.** The most highly expressed 47 genes and their RPKM values in the six samples. (XLS 24 kb)

Additional file 6: **Table S5.** DE genes with the FDR value≤0.001 and the absolute value of log 2 Ratio ≥ 1. Gene Length, length of all exon in gene; Expression, reads number that uniquely mapped to gene; *P* value, *p* value for hypothesis testing. (XLS 2504 kb)

Additional file 7: **Table S6.** GO analysis of DE genes with corrected *P* value ≤0.05. The letter “n” is number of DE genes which are annotated to this ontology. The letter “m” is number of DE genes which are annotated to this term. (XLS 789 kb)

Additional file 8: **Table S7.** KEGG pathway analysis of DE genes with Q value ≤1. Significantly enriched biological pathways were identified in DE genes based on the entire genome background. Q value is a *P* value that has been adjusted for the FDR value. Pathways with Q value ≤0.05 are thought to be significantly enriched. (XLS 105 kb)

## Abbreviations

Co-genes: Common highly expressed genes
DE genes: Differentially expressed genes
GLT: Guangling large tailed
GO: Gene ontology
KEGG: Kyoto encyclopedia of genes and genomes
PEF: Perirenal fat
qRT-PCR: Quantitative real-time PCR
RNA-seq: RNA sequencing
RPKM: Reads per kilobase of exon model in a gene per million mapped reads
STH: Small tailed han
SUF: Subcutaneous fat
TAF: Tail fat
