# Supplementary figures and images for: Transcriptome analysis of adipose tissues from two fat-tailed sheep breeds reveals key genes involved in fat deposition
Source: BMC Genomics. 2018 May 8;19:338. doi: 10.1186/s12864-018-4747-1 (PMC5941690; doi:10.1186/s12864-018-4747-1)

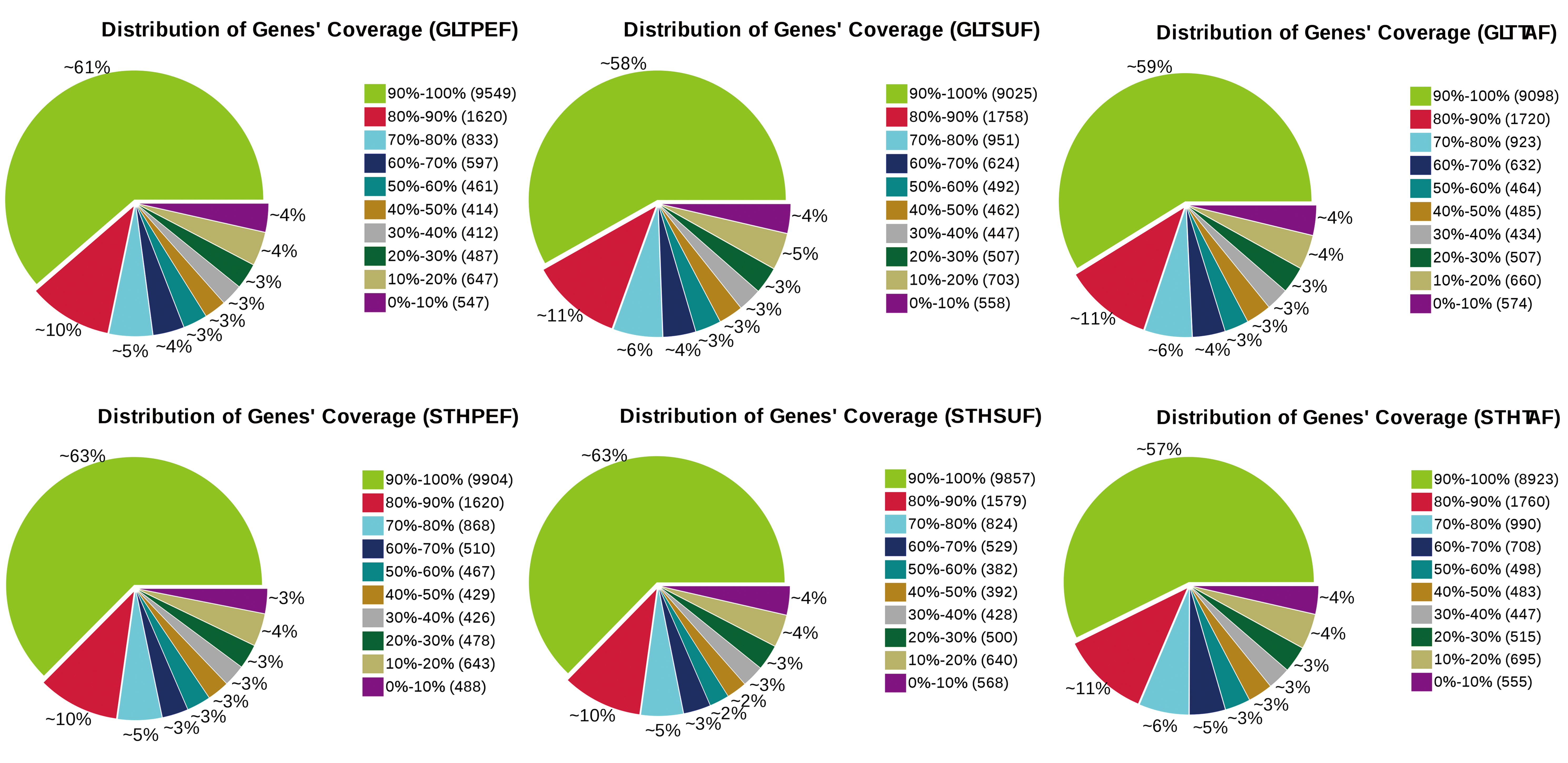

Supplement: Supplementary file 2 — Figure S1. Gene coverage distribution of the six adipose tissue transcriptomes. Gene coverage is calculated as the percentage of a gene covered by reads. This value is equal to the ratio of the base number in a gene covered by unique mapping reads to the total base number of coding region in that gene. Pies with different colors represent proportions of genes with certain coverage. For example, green pie is indicating proportion of genes with coverage between 90 and 100%. (TIF 7235 kb) [file 12864_2018_4747_MOESM2_ESM.tif]
